# Accuracy of Cone-beam Computed Tomography in Comparison with Standard Method in Evaluating Root Canal Morphology: An *In Vitro* Study

**DOI:** 10.22037/iej.v13i2.18614

**Published:** 2018

**Authors:** Zahra Dalili Kajan, Mehran Taramsari, Negar Khosravi Fard, Mohsen Kanani

**Affiliations:** a *Dental Sciences Research Center, Department of Maxillofacial Radiology, Dental School, Guilan University of Medical Sciences, Rasht, Iran; *; b * Dental Sciences Research Center, Department of Endodontics, Dental School, Guilan University of Medical Sciences, Rasht, Iran; *; c * Department of Maxillofacial Radiology, Dental School, Guilan University of Medical Sciences, Rasht, Iran; *; d * Dentist, Private Office, Dental School, Guilan University of Medical Sciences, Rasht, Iran*

**Keywords:** Anatomy, Cone-beam Computed Tomography, Root Canals

## Abstract

**Introduction:**

In order to successfully perform root canal treatment, thorough knowledge of the root canal anatomy is essential. Cone-beam computed tomography (CBCT) has the ability to improve our understanding of the root canal system. The goal of the present study was to compare the accuracy of CBCT in revealing the number and form of the root canals of different maxillary and mandibular teeth with clearing and staining method.

**Methods and Materials:**

CBCT images were taken from 80 extracted human teeth fixed in agar arch models. The number and configuration of the root canals of each tooth were determined by the two observers. Then the teeth were cleared and stained. Two endodontists evaluated the number and forms of the root canals. The accuracy of CBCT was determined and compared with clearing and staining by Fisher’s exact test. The agreement of two methods in detection of the number and form of the root canals were evaluated by Kappa test, *P*≤0.05.

**Results:**

CBCT accurately detected the number of root canals in 129 (92.1%) of 140 roots and the form of the canals in 119 (85%) of the roots. There was no significant difference between the accuracy of CBCT in the detection of the number (*P*=0.13) and forms (*P*=0.4) of root canals of maxillary and mandibular teeth. The agreement between CBCT, and tooth clearing and staining in detection of the number of root canals was excellent in the maxilla (kappa=0.88±0.05) and good in the mandible (kappa=0.720±0.097). The agreement between the two methods in demonstration of the form of root canals was good in both maxillary (kappa=0.73±0.07) and mandibular (kappa=0.67±0.09) teeth.

**Conclusion:**

CBCT provides accurate information about root canal morphology. Application of this technique could result in more successful endodontic treatments.

## Introduction

Successful completion of root canal treatment requires accurate knowledge of internal root morphology and its possible variations, which directly influence the quality of debridement, disinfection, and obturation of the root canal system [1-6]. The number and morphology of the root canals vary according to age, sex, and ethnicity [1, 7-10]. Furthermore, it has been postulated that the complexities of the root canal system are determined genetically and therefore should be considered among different populations [2, 3]. 

Various methods have been applied for analysis of the root canal morphology, of which the most common are canal staining and tooth clearing [2, 3, 10-12], conventional radiography [13, 14], digital radiographic techniques [15-17], and radiographic assessment enhanced with contrast media [18, 19]. More recently, cone-beam computed tomography (CBCT) [20], modified canal staining and tooth clearing [21] and micro-computed tomography (micro-CT) [22-24] have been used. The most frequently used *in vivo* methods among the abovementioned techniques are periapical radiography and CBCT. Periapical radiographs can be misleading for the assessment of root anatomy due to the superimposition of the roots or their surrounding structures [1]. In contrast, numerous studies have used CBCT as an accurate method for evaluating the root canal system [25-27].

**Figure 1:**
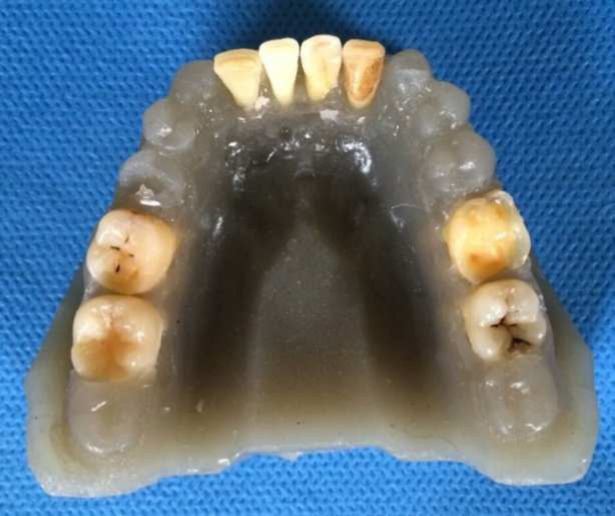
Agar model of mandible for CBCT evaluation

CBCT was introduced to the field of endodontics in 1990. The technique uses a cone-shaped source of radiation to acquire image data in a full or half arc of rotation, which displays the three-dimensional outline of an object, thereby enabling the clinician to achieve a more realistic representation of the structures being imaged. The ability of CBCT to reduce or eliminate the superimposition of the surrounding structures makes it superior to periapical radiographs [27]. Blattner *et al.* [25] evaluated the presence of second mesiobuccal (MB2) canal in maxillary first and second molar teeth using CBCT and concluded that this technique is a reliable method of detecting additional canals when compared with the gold standard of physical sectioning.

Oridenla-Zapata *et al.* [24] in a comparative study on accuracy of three different systems; micro-CT, CBCT and the clearing method reveals that CBCT and clearing method were not accurate method for evaluating the actual root anatomy but this study is only limited to mesial root of mandibular first molar. 

To the best of our knowledge the accuracy of CBCT in comparing peripheral quantitative computed tomography, spiral computed tomography (SCT), plain, and contrast medium-enhanced digital radiographs in evaluating the root canal morphology was studied in one study [28] but it was used widespread in this kind of epidemiological evaluation. 

The present study was designed to compare CBCT in revealing the number and form of the root canals of maxillary and mandibular teeth in comparison with the gold standard of tooth clearing and staining. The most important questions are “what is the accuracy of CBCT for studying the root canal morphology?” and “is it comparable with reliable method of clearing and staining in this kind of endodontic evaluations?”

## Materials and Methods

***Sample preparation***

Eighty extracted human teeth, including 20 first or second mandibular molars (8 mandibular first and 12 mandibular second molars), 20 mandibular central or lateral incisors (10 of each teeth), 20 first or second maxillary molars (13 maxillary first, 7 maxillary second molars), and 20 single-rooted maxillary premolars, were selected randomly from the collected samples in department of maxillofacial surgery, Guilan University of Medical Sciences. The teeth were stored in Savlon antiseptic solution. The Exclusion criteria for tooth selection were root caries, root crack and severe calcified canal. Calcification of the canals were evaluated in periapical radiographs by an endodontist that was not as invited as observers of CBCT or cleared and stained samples. The examiners were blinded to the anatomy of the pulp canals. The teeth were randomly arranged in 10 agar jaw models (Figure 1). Each jaw contained eight teeth that included either four mandibular molars and four mandibular incisors or four maxillary molars and four maxillary premolars. The teeth were fixed in place with wax. Agar was selected due to its ability to simulate soft tissues surrounding the teeth. This study was confirmed by Ethic committee of Guilan University of Medical Sciences; IR.GUMS.REC.1394.514. “All procedures followed were in accordance with the ethical standards of the responsible committee on human experimentation (Guilan University of Medical Sciences) and with the Helsinki Declaration of 1975, as revised in 2008”.

***Imaging examinations***

Each jaw model was imaged by a CBCT device (NewTom, Verona, Italy) with the exposure parameters of 110 kV, 0.5 mA and 2.04 mA. The 4-inch field of view (FOV) (0.200-0.240 mm voxel size) in standard scan mode was selected in imaging protocol. The volumetric data set of each imaging procedure was converted into study images *via* NNT software (NewTom, Verona, Italy). A maxillofacial radiologist and an endodontist both with over 10 years of professional experience evaluated the axial and cross-sectional image slices (1 mm thickness at 0.5 mm intervals). The observers of imaging examination section were different from the observers of tooth clearing and staining section. The observers’ expertise were very important in each section. The number and configuration of the root canals of each tooth were determined by the two observers. Canal types were determined according to Vertucci’s classification. [29] CBCT images of 20 teeth were randomly selected and re-evaluated by the observers to evaluate the intra-examiner reliability or the inter-observer agreement. 

***Tooth clearing and staining***

**Figure 2:**
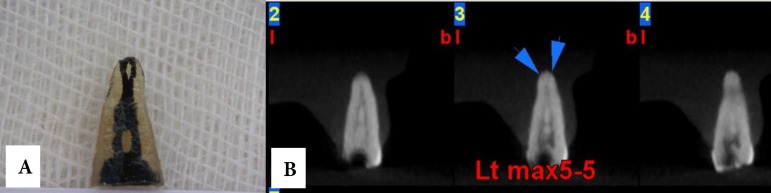
*A) *A maxillary premolar tooth after being cleared and stained; *B)* Cross-sectional CBCT images of the same tooth within the agar model

**Figure 3:**
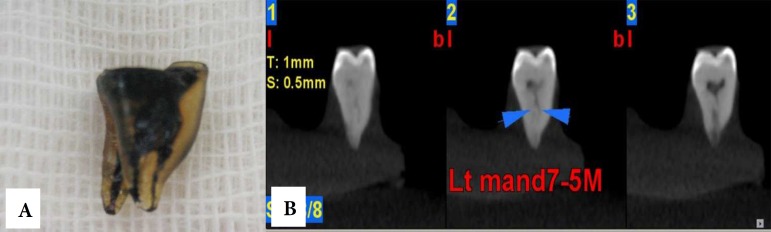
*A)* A mandibular molar tooth after clearing and staining; *B)* Axial and cross-sectional CBCT images of the mesial root of the same tooth fixed in an agar model

After imaging, the teeth were taken out of the agar models and prepared for clearing and staining. First, the root surfaces were debrided with a curette. The crowns of the teeth were cut at the level of the cemento-enamel junction. The teeth were placed in a 5.25% sodium hypochlorite solution for 24 h to eliminate any residual pulp tissue. The teeth were rinsed with water for 1 h and then dried. Then, the samples were placed in 5% nitric acid for 72 h. The solution was agitated every 8 h and renewed every 24 h. After 72 h, the samples were rinsed with water for 4 h. After rinsing, the teeth were dehydrated with 80% ethylic alcohol for 12 h, 90% ethylic alcohol for 1 h, and 100% ethylic alcohol for 1 h (in that order). Liquid ink (Pelikan, Iran) was injected into the canals with a 27-gauge needle and suctioned apically until seen throughout the apical foramen. Finally, the samples were placed in 99% methyl salicylate. Once the teeth were cleared and stained, two endodontists evaluated the number and forms of the root canals by observation based on Vertucci classification. Using of magnifier by observers was optional. Examples of CBCT images of the two teeth as well as their appearances after clearing and staining are shown in Figures 2 and 3.

All data were analyzed with SPSS software (SPSS version 22, SPSS, SAS, Chicago, IL, USA). Fisher’s exact test was used to assess the frequency of correct detection of the number and form of root canals by CBCT. The strength of agreement between the results obtained from CBCT and tooth clearing and staining was determined *via *the Kappa test. The level of significance was set at 0.05. 

## Results

In the current study, a total of 140 roots were evaluated with respect to the ability of CBCT to reveal the number and form of the pulp canals. The intra-examiner reliability in evaluation of CBCT images was calculated to be 91%. CBCT accurately detected the number of pulp canals in 129 (92.1%) roots. With respect to the configuration of root canals, 119 (85%) roots were correctly demonstrated by CBCT; however, the forms of root canals were not properly visualized in 21 (15%) roots. Table 1 presents the frequency of correct diagnosis of the number(s) and forms of the root canals by CBCT in maxillary and mandibular teeth. The frequency of correct diagnosis of the number(s) and form(s) of the root canals according to the tooth type and root type is presented in Tables 2 and 3, respectively. Detectability of the number and form of the root canals was uninfluenced by the type of jaw, tooth, or root. 

The agreement between CBCT and tooth clearing and staining in detecting the number and revealing the form of the root canals was determined by Kappa agreement test. The strength of agreement is divided to poor (<0.2), fair (0.21-0.4), moderate (0.41-0.6), good (0.61-0.8) and excellent (0.81-1). The agreement between CBCT, and tooth clearing and staining in detection of the number of root canals was excellent in the maxilla (kappa=0.88±0.05) and good in the mandible (kappa=0.720±0.097) by considering the *P*-value of 0.001. The agreement between the two methods in demonstration of the form of root canals was good in both maxillary (kappa=0.73±0.07) and mandibular (kappa=0.67±0.09) teeth. The *P*-value was 0.001. The agreement between CBCT and tooth clearing and staining in detecting the number and revealing the form of the root canals based on the tooth type was presented in Table 4.

**Table 1:** Frequency of correct diagnosis of the number and form of the root canals in maxillary and mandibular teeth by cone beam computed tomography (CBCT)

**Tooth**	**Correct diagnosis**	
	**Number of root canals**	**Form of root canals**
**Maxillary teeth**	76 (95%)	69 (86.3%)
**Mandibular teeth**	53 (88.3%)	50 (83.3%)
***P*** **-value** *	0.13	0.4

* *Fisher’s exact test, P≤0.05*

**Table 2:** Frequency of correct diagnosis of the number and form of the root canals by CBCT according to tooth type

		**Correct diagnosis**			
**Jaw**	**Tooth type**	**Number of root canals**	***P*** -value*	**Form of root canals**	***P*** **-value** *
**Maxilla**	**Molar teeth**	57 (95%)	0.69	51 (85%)	0.45
	**Premolar teeth**	19 (95%)		18 (90%)	
**Mandible**	**Molar teeth**	36 (90%)	0.43	33 (82.5%)	0.56
	**Incisor teeth**	17 (85%)		17 (85%)	

* *Fisher’s exact test, P≤0.05*

## Discussion

In the present study, we evaluated the accuracy of CBCT in detecting the number and revealing the form of root canals and compared the results with those of the gold standard tooth clearing and staining method. Maxillary molar and premolar teeth as well as mandibular molar and incisor teeth were selected as study samples since these teeth have the greatest amount of variation in the features of the root canal system. 

In very similar study to ours; Ordinola-Zapata *et al.* [24] revealed that type I of root form in mesial root of mandibular first molar in clearing method was detected lower than CBCT and micro-CT but in type II, there was no difference. Although, the clearing method has the limitation in flowing laterally into fine anatomical root structures such as isthmii but it could not explain the Ordinola-Zapata *et al.* [24] findings. The type I root form could not have above-mentioned limitation in flowing the dye. The CBCT machine used in our study has 360 degree rotation around the object that is comparable with the degree of rotation in micro-CT thus the details of root canal space defined more accurately. In the study by Ordinola-Zapata *et al.* [24] the device was Palanmeca (proMax 3Ds) with 200 degree rotation around the object.

With respect to the detection of root canals, CBCT was successful in 129 of 140 (92.1%) roots; however, CBCT did not accurately detect the number of root canals in 11 (7.9%) roots of teeth, of which seven teeth belonged to the mandible (four molars and three incisors) and four belonged to the maxilla (three molars and one premolar).

The percentage of correct diagnoses of the number of root canals with CBCT in this study was 92.1%, which was comparable with the result reported by Neelakantan *et al.* [28] (99%) but much higher than the findings reported by Blattner *et al.* [25] (78.95%). This discrepancy may possibly be explained by the differences in the sample sizes, types of CBCT devices, and thickness of the image slices. Michetti *et al.* [30] showed a high correlation between CBCT and histological sections but they studied only nine extracted teeth. 

With respect to tooth type, CBCT correctly diagnosed the number of root canals in 36 of 40 mandibular molar roots, 17 of 20 mandibular incisor roots, 57 of 60 maxillary molar roots, and 19 of 20 maxillary premolar roots. These results were not statistically significant; in other words, tooth type was not an influential factor in the accuracy of CBCT for detecting the number of canals. This was comparable to the results reported by Neelakantan *et al.* [28]. 

**Table 3:** Frequency of correct diagnosis of the number and form of the root canals by CBCT according to root type in multi-rooted teeth

**Tooth**	**Root type**	**Correct diagnosis**			
		**Number of root canals**	***P*** -value*	**Form of root canals**	***P*** **-value** *
**Maxillary molars**	**Mesiobuccal**	18 (90%)	0.76	13 (65%)	0.07
	**Distobuccal**	19 (95%)		18 (90%)	
	**Palatal**	20 (100%)		20 (100%)	
**Mandibular molars**	**Mesial**	18 (90%)	0.99	15 (75%)	0.48
	**Distal**	17 (89.5%)		17 (89.5%)	

* *Fisher’s exact test*

**Table 4:** Agreement of CBCT and tooth clearing and staining in demonstrating the number and form of the root canals according to tooth type

**Jaw**	**Tooth type**	**Number of root canals**	***P*** **-value**	**Form of root canals**	***P*** **-value**
		**kappa values(SE)** *		**kappa values(SE)**	
**Maxilla**	**Molar teeth**	0.85 (0.08)	0.0001	0.63 (0.09)	0.0001
	**Premolar teeth**	0.89 (0.10)	0.0001	0.83 (0.11)	0.0001
**Mandible**	**Molar teeth**	0.77 (0.10)	0.0001	0.67 (0.10)	0.0001
	**Incisor teeth**	0.58 (0.20)	0.004	0.65 (0.17)	0.0001

* *SE; standard error*

Neelakantan *et al.* [28] assessed the accuracy of various techniques for evaluation of the root and canal morphology in extracted human teeth. They concluded that CBCT did not differ greatly from the gold standard of tooth clearing and staining. Of interest, the accuracy of CBCT was shown to be higher than that of spiral CT and plain digital radiography [28]. Mittachi *et al.* [30] and Fernandes *et al.* [31] reported that CBCT could be successful method in evaluation of root form. Fernandes *et al. *[31] evaluated three CBCT machine and digital periapical radiographs in evaluation of forty mandibular incisors in comparing with micro-CT. There was a high degree of accuracy for all selected methods about type I and type Ia root canal as well as type III. The oval canals were identified by the NewTom CBCT device in this study.

In the present study, the anatomical location of the tooth root (mesial or distal in mandibular molars, and mesiobuccal, distobuccal, or palatal in maxillary molars) did not considerably influence the detection of root canals.

In an *in vitro* study, Blattner *et al.* [25] evaluated the accuracy of CBCT in revealing the number of root canals and, specifically, MB2 canals in maxillary molars. They reported that the results obtained from CBCT examinations were strongly consistent with the results obtained using the gold standard of the study, which was tooth sectioning [25]. Similar results were noticed from Domark *et al.* study [32] in comparison between CBCT and micro-CT. 

With respect to delineating the root canal forms in the current study, CBCT ability was not influenced by jaw and root types, although tooth type had a small effect. CBCT was acceptably accurate in demonstrating the form of root canals in most tooth types, but was less accurate in the mandibular incisors. The small diameter of the incisor roots in the mandible and low contrast of the image of the root canal against the thickness of the root could be contributing factors for the lower level of accuracy of CBCT in revealing the root canal forms. 

The agreement of CBCT and tooth clearing and staining in the detection of the number of root canals was excellent in the maxillary and good in the mandibular teeth. The lower agreement of the results in the mandibular teeth is perhaps due to the intermediate conformity of CBCT and tooth clearing in the mandibular incisors. CBCT and tooth clearing methods also showed good to excellent levels of agreement in displaying the root canal forms in both maxillary and mandibular teeth.

Although the agreement between CBCT and tooth clearing results was not flawless, the accuracy of CBCT in revealing both the number and form of the root canals was convincing enough to be considered as an additional diagnostic tool for the evaluation of the root canal system in the field of endodontics. Likewise, Matherne *et al.* [26] and Baratto *et al.* [14] pointed out the impact of CBCT on the improvement of the root canal morphology evaluation.

As most of the previously performed studies were limited to an evaluation of a specific root canal of the maxillary molar teeth, a broad comparison of our results with those of the available literature could not be conducted. 

## Conclusion

The present research revealed that CBCT displays root canal morphology with high accuracy owing to the creation of multi-planar images of the root structures. This technique is therefore recommended for improving the clinician’s knowledge of the root canal morphology, which will undoubtedly result in more successful endodontic treatments.
